# Effect of Dietary Zinc Oxide on Morphological Characteristics, Mucin Composition and Gene Expression in the Colon of Weaned Piglets

**DOI:** 10.1371/journal.pone.0091091

**Published:** 2014-03-07

**Authors:** Ping Liu, Robert Pieper, Juliane Rieger, Wilfried Vahjen, Roger Davin, Johanna Plendl, Wilfried Meyer, Jürgen Zentek

**Affiliations:** 1 Institute of Animal Nutrition, Department of Veterinary Medicine, Freie Universität Berlin, Berlin, Germany; 2 Institute of Veterinary Anatomy, Department of Veterinary Medicine, Freie Universität Berlin, Berlin, Germany; 3 Grup de Nutrició, Maneig i Benestar Animal, Departament de Ciència Animal i dels Aliment, Facultat de Veterinària, Universitat Autònoma de Barcelona, Barcelona, Spain; 4 Institute of Anatomy, University of Veterinary Medicine Hannover Foundation, Hannover, Germany; Louisiana State University School of Veterinary, United States of America

## Abstract

The trace element zinc is often used in the diet of weaned piglets, as high doses have resulted in positive effects on intestinal health. However, the majority of previous studies evaluated zinc supplementations for a short period only and focused on the small intestine. The hypothesis of the present study was that low, medium and high levels of dietary zinc (57, 164 and 2,425 mg Zn/kg from zinc oxide) would affect colonic morphology and innate host defense mechanisms across 4 weeks post-weaning. Histological examinations were conducted regarding the colonic morphology and neutral, acidic, sialylated and sulphated mucins. The mRNA expression levels of mucin (*MUC*) 1, 2, 13, 20, toll-like receptor (*TLR*) 2, 4, interleukin (*IL*)-1β, 8, 10, interferon-γ (*IFN-γ*) and transforming growth factor-β (*TGF-β*) were also measured. The colonic crypt area increased in an age-depending manner, and the greatest area was found with medium concentration of dietary zinc. With the high concentration of dietary zinc, the number of goblet cells containing mixed neutral-acidic mucins and total mucins increased. Sialomucin containing goblet cells increased age-dependently. The expression of *MUC2* increased with age and reached the highest level at 47 days of age. The expression levels of *TLR2* and *4* decreased with age. The mRNA expression of *TLR4* and the pro-inflammatory cytokine *IL-8* were down-regulated with high dietary zinc treatment, while piglets fed with medium dietary zinc had the highest expression. It is concluded that dietary zinc level had a clear impact on colonic morphology, mucin profiles and immunological traits in piglets after weaning. Those changes might support local defense mechanisms and affect colonic physiology and contribute to the reported reduction of post-weaning diarrhea.

## Introduction

For reasons of health prophylaxis and growth-promoting purpose, so-called pharmacological levels of zinc oxide are often used in the feeding of weaning piglets. The beneficial effects of the trace element zinc on the prophylaxis and treatment of diarrhea are well documented both in pigs [1]–[3] as well as in other species including humans [4]. Zinc has been shown to have a positive impact on daily gain in the post-weaning period of piglets when administered at so-called pharmacological concentrations [5]. Zinc has multiple effects on physiological and pathophysiological processes, including the diversity and metabolic activity of the intestinal microbiota [6]–[10], the gut associated immune system [11] and absorptive and secretory processes [1], [12], [13].

Previous studies showed that high alimentary zinc intakes lead to significantly increasing concentrations of zinc in the digesta of the gastrointestinal tract, particularly in the colon. Pigs fed 2000 mg additional zinc as ZnO per kg complete diet were shown to have 600 mg zinc per kg digesta retained in the ileum, which enriched to 2141 mg zinc per kg digesta in the colon [14]. Such high concentrations of zinc can be expected to induce a broad spectrum of reactions in the gut tissue. Despite well-established counter-regulatory mechanisms of the zinc transporters in the intestine in response to high dietary intakes [15], there is a time-dependent accumulation of high quantities of zinc in a variety of tissues [16], [17]. Zinc accumulation has been shown in the bone, and other tissues such as the liver. The intestinal mucosa is of specific interest. It is the primary contact site between digesta and the host organism and might react in a specific manner. It has not been studied if and to which extent elevated concentrations of zinc in the digesta induce morphological changes or affect inflammatory parameters in the colon of piglets. Pigs that have been fed diets with high zinc levels for different lengths of time might have different reaction patterns concerning pro-and anti-inflammatory cytokines, but this has not been studied in detail.

Therefore, the aim of the present study was to evaluate the impact of three concentrations of dietary zinc over four weeks after weaning on morphological parameters, mucin composition and gene expression related to innate immunity and inflammatory response in colonic tissue of young piglets.

## Materials and Methods

This study involving pig handling and treatments was carried out in accordance with German animal welfare law. The protocol was approved by State Office of Health and Social Affairs Berlin (LaGeSo Reg. Nr. 0347/09).

### Animals, Housing and Diets

A total of 96 purebred Landrace piglets was weaned at the age of 26±1 d (mean BW: 7.5±1.2 kg) and randomly allocated into three groups balancing for litter and gender. Piglets were housed in commercial flat-deck pens (2 piglets per pen) with stainless steel framings. Room temperature was maintained at 26°C on the day of weaning and reduced at regular intervals to achieve 22°C one week post-weaning. The humidity was kept constant and the lightning program was maintained at 16 h light and 8 h dark per day. Water and feed were provided ad libitum. From 12 days of age, piglets were provided a non-medicated pre-starter diet. After weaning, piglets received a mash starter diet until 54 days of age. Piglets in each group received one of three experimental diets (Table 1) based on wheat, barley, and soybean meal. The analyzed zinc concentration in the basal diet was 35 mg/kg and the target zinc concentrations of the three diets were adjusted by replacing corn starch with analytical-grade zinc oxide (Sigma). The final zinc levels were adjusted to low (57 mg/kg zinc), medium (164 mg/kg zinc), and high (2425 mg/kg zinc) dietary zinc, respectively.

**Table 1:** Ingredients and analyzed chemical composition of diets.

Ingredients (g/kg as feed)		Analyzed chemical composition	
Wheat	380	Dry matter (g/kg fresh matter)	879
Barley	300	Crude ash (g/kg DM)	81
Soybean meal	232	Crudeprotein (g/kg DM)	194
Corn starch/zinc oxide^1^	10	Crudefiber (g/kg DM)	36
Limestone	20	Ether extract (g/kg DM)	34
Monocalcium phosphate	20	Starch (g/kg DM)	376
Mineral & Vitamin Premix^2^	15	Lysine (g/kg DM)	11.7
Soy oil	17.5	Methionine (g/kg DM)	4.0
Salt	2.0	Threonine (g/kg DM)	7.2
Lysine HCl	2.5	Tryptophan (g/kg DM)	2.4
Methionine	1.0	Calcium (g/kg DM)	11.0
		Phosphorus (g/kg DM)	8.0
		Sodium (g/kg DM)	3.1
		Magnesium (g/kg DM)	2.2
		Zinc^3^ (mg/kg DM)	34
		Iron (mg/kg DM)	309
		Manganese (mg/kg DM)	41
		Copper (mg/kg DM)	7
		Metabolisable energy (MJ/kg)	13.0

1 Corn starch in the basal diet was partially replaced with analytical grade zinc oxide (Sigma Aldrich, Taufkirchen, Germany) to adjust for the zinc concentration.

2 Mineral and Vitamin Premix (SpezialfutterNeuruppin Ltd., Neuruppin, Germany), providing per kg feed: 1.95 g Na (sodium chloride), 0.83 g Mg (magnesium oxide), 10,500 IU vitamin A, 1,800 IU vitamin D3, 120 mg vitamin E, 4.5 mg Vitamin K_3_, 3.75 mg thiamine, 3.75 mg riboflavin, 6.0 mg pyridoxine, 30 µg cobalamine, 37.5 mg nicotinic acid, 1.5 mg folic acid, 375 µg biotin, 15 mg pantothenic acid, 1,200 mg choline chloride, 75 mg Fe (iron-(II)-carbonate), 15 mg Cu (copper-(II)-sulfate), 90 mg Mn (manganese-(II)-oxide), 675 µg I (calcium-iodate), 525 µg Se (sodium-selenite).

3 Analyzed concentration of zinc in the basal diet without ZnO supplementation. The diets as fed contained 57, 164, and 2425 mg zinc/kg, adjusted by zinc oxide.

### Sampling

Randomly selected piglets (n = 8 per dietary treatment and time point, respectively) were euthanized at 33±1, 40±1, 47±1 and 54±1 d of age balancing for litter and gender, resulting in a duration of dietary treatment of 1, 2, 3, and 4 weeks, respectively. Piglets were anesthetized with 20 mg/kg BW of ketamine hydrochloride (Ursotamin®, SerumwerkBernburg AG, Germany) and 2 mg/kg BW of azaperone (Stresnil®, Jansen-Cilag, Neuss, Germany) prior to euthanization by intracardial injection of 10 mg/kg BW of tetracaine hydrochloride, mebezonium iodide and embutramide (T61®, Intervet, Unterschleißheim, Germany). The gastrointestinal tract was removed and the small intestine was separated from the large intestine and the mesentery. A 3 cm long segment of ascending colon was longitudinally cut along mesenteric attachment and rinsed with PBS buffer, and then the tissue was pinned on cork and fixed immediately in Bouin’s solution for histological examinations. Another 5 cm segment of colonic tissue was snap-frozen in liquid nitrogen and stored at −80°C until mRNA extraction and gene expression analysis performed.

### Histochemistry on the Colon

Samples from ascending colon were fixed in Bouin’s solution for three days, and then rinsed several times in 70% ethanol to remove the remaining picric acid, dehydrated with a graded series of ethanol, and cleaned with xylol. The samples were embedded in paraffin wax according to standard protocols [18]. Five µm sections were cut from the paraffin blocks using a rotary microtome, and sections dried at 37°C in an incubator. Then the slides with sections were deparaffinized with xylene, and rehydrated in a series of descending ethanol for subsequent staining procedures.

Different staining methods were applied in order to distinguish different mucin chemotypes characterized by the specific carbohydrates present on their surfaces. The Alcian blue pH 2.5-periodic acid Schiff (AB-PAS) (AB-8GX, Sigma; Schiff’s reagent, Merck, Darmstadt, Germany) staining procedure [19] was carried out to distinguish neutral and acidic mucin in goblet cells. The neutral and acidic mucins in goblet cells were stained in magenta and blue colors, respectively. The mixture of neutral-acidic mucins showed purple, magenta-purple or blue-purple colors in goblet cells. To identify sialomucins and sulfomucins, tissues were stained with the high iron diamine-Alcian blue pH 2.5 (HID-AB) technique, including counterstaining with nuclear fast red [20]. As a result, sulfomucins were stained black, sialomucins were stained blue, and a mixture of sulfo-sialomucins resulted in black and blue colors. For the quantification of the cells producing different mucin chemotypes, ten well-defined colonic crypts were randomly selected from different sections in each animal stained with AB-PAS and HID-AB methods respectively. Goblet cells producing various mucins were classified and quantified in crypts. The corresponding basement membrane length in the colonic crypts was used as reference, and the number of the goblet cells with different mucins was expressed as quantity in per 1 mm basement membrane length.

The colonic tissues stained with AB-PAS were further used for the morphological measurements. Crypt depth and area were measured in ten well-orientated crypts in each animal. Crypt depth was measured as the distance from the crypt base at the basement membrane to the crypt mouth. The area was determined on the same crypts as the area encircled by the basement membrane and crypt mouth including the crypt lumen [21]. All the determination was carried out using a light microscope (Zeiss Photomicroscope III, Oberkochen, Germany) connected with a digital camera (Olympus DP72, Japan) and analyzed for morphometric characteristics with cellSens standard software (Olympus, version 1.4).

### RNA Extraction and Gene Expression in Colon by Real-Time PCR

RNA extraction and analysis of colonic tissue gene expression were accomplished as described [22]. The quality and quantity of mRNA was determined with a Bioanalyzer (Agilent 2100, Waldbronn, Germany). The expression of the following target genes was analyzed: mucin (*MUC*) 1, 2 13, 20, toll-like receptor (*TLR*) 2, 4, interleukin (*IL*)-1β, 8, 10, interferon-γ (*IFN-γ*), transforming growth factor-β (*TGF-β*). The 60S ribosomal protein of L19 (*RPL19*), *RPL13* and β-2-microglobulin were used as housekeeping genes for data normalization (Table 2). The Ct values were normalized using the mean values of the housekeeping genes and arbitrary values were calculated and used for statistical comparisons. Melting curves and PCR efficiency were used as standard quality criteria for each RT-PCR run.

**Table 2:** PCR primers used for gene expression analysis.

Target	Sequences of primers (5′ to 3′)	A_T_	Reference
RPL19	GCTTGCCTCCAGTGTCCTC	60	Pieper et al., 2012
	GCGTTGGCGATTTCATTAG		
RPL13	CCGTCTCAAGGTGTTCGATG	60	This study
	GGATCTTGGCCTTCTCCTTC		
B2M	CCCCGAAGGTTCAGGTTTAC	60	Martin et al., 2013
	CGGCAGCTATACTGATCCAC		
MUC1	GGTACCCGGCTGGGGCATTG	60	Pieper et al., 2012
	GGTAGGCATCCCGGGTCGGA		
MUC2	CTGCTCCGGGTCCTGTGGGA	60	Pieper et al., 2012
	CCCGCTGGCTGGTGCGATAC		
MUC13	GCTACAGTGGAGTTGGCTGT	60	This study
	GACGAATGCAATCACCAGGC		
MUC20	GAAGGGGGCATCGCTGCCTG	60	Pieper et al., 2012
	GCCAGGGTCCCACTGCCATG		
TLR2	CACGTGCTGATGGAGGGGCAT	60	This study
	GCCCAATGACGCCTCGGTGAT		
TLR4	AGGCCGTCATTAGTGCGTCAGT	60	This study
	AGCCCACAAAAAGCAACAAGTGGA		
IL-1β	TGAAGTGCCGCACCCAAAACCT	60	Pieper et al., 2012
	CGGCTCCTCCTTTGCCACAATCA		
IL-8	GGTCTGCCTGGACCCCAAGGAA	60	This study
	TGGGAGCCACGGAGAATGGGTT		
IL-10	GTCCGACTCAACGAAGAAGG	60	Pieper et al., 2012
	GCCAGGAAGATCAGGCAATA		
IFN-γ	TCCAGCGCAAAGCCATCAGTG	58	This study
	ATGCTCTCTGGCCTTGGAACATAGT		
TGF-β	AGAAGCAGAGGGTGGGAAAT	60	Pieper et al., 2012
	AAGAAGGCGAGAGGAGGAAC		

A_T_: annealing temperature; RPL19∶60S ribosomal protein L19; RPL13∶60S ribosomal protein L13; B2M: beta-2 microglobulin; MUC1: mucin 1; MUC2: mucin 2; MUC13: mucin 13; MUC20: mucin 20; TLR2: toll-like receptor 2; TLR4: toll-like receptor 4; IL-1β: interleukin 1β; IL-8: interleukin 8; IL-10: interleukin 10; IFN-γ: interferon-γ; TGF-β: transforming growth factor-β.

### Statistical Analysis

Statistical analysis was performed using SPSS 19.0 (Chicago, IL, USA). The data on the colonic morphology, mucin chemotypes in goblet cells and gene expression related to innate immunity and inflammatory cytokines were tested for normal distribution by Shapiro Wilk test. They were analyzed using two-way analysis of variance (ANOVA) followed by Tukey post hoc test with diet and age as fixed factors, and data were given as mean values. Differences were considered significant at *P*<0.05.

## Results

### Morphometric Measurements in the Colon

The colonic crypt depth was not affected by the dietary zinc concentration or age (Table 3), whereas the crypt area increased age-dependently (*P* = 0.001). The highest values were found in the medium dietary zinc group (*P* = 0.003).

**Table 3:** Morphometric characteristics in the ascending colon of weaned piglets^1^.

Age	33 d			40 d			47 d			54 d				*P*-value		
Diet	LZn	MZn	HZn	LZn	MZn	HZn	LZn	MZn	HZn	LZn	MZn	HZn	SEM	Age	Diet	Age×Diet
CD (µm)	338	341	320	357	347	344	361	362	335	358	379	347	4.01	0.102	0.089	0.927
CA (µm^2^)	18267^ab^	19892^ab^	16609^b^	20577^ab^	18242^ab^	18152^ab^	20700^ab^	22270^a^	19317^ab^	20672^ab^	21811^a^	19729^ab^	252	0.001	0.003	0.255

1 Ninety-six 26 day weaned Landrace piglets were randomly allocated into three diets with low, medium and high dietary zinc (57, 164, and 2425 mg/kg diet from ZnO). Eight piglets in each group were killed at the age of 33±1, 40±1, 47±1 and 54±1 d. Data were analyzed by two-way ANOVA followed by Tukey post hoc test. The data were given as mean values.

LZn: low dietary zinc; MZn: medium dietary zinc; HZn: high dietary zinc; CD: crypt depth; CA: crypt area.

ab Means with different superscripts within a row indicate significant differences between groups (*P*<0.05).

### Mucin Chemotypes in Colonic Goblet Cells

**Figure 1:**
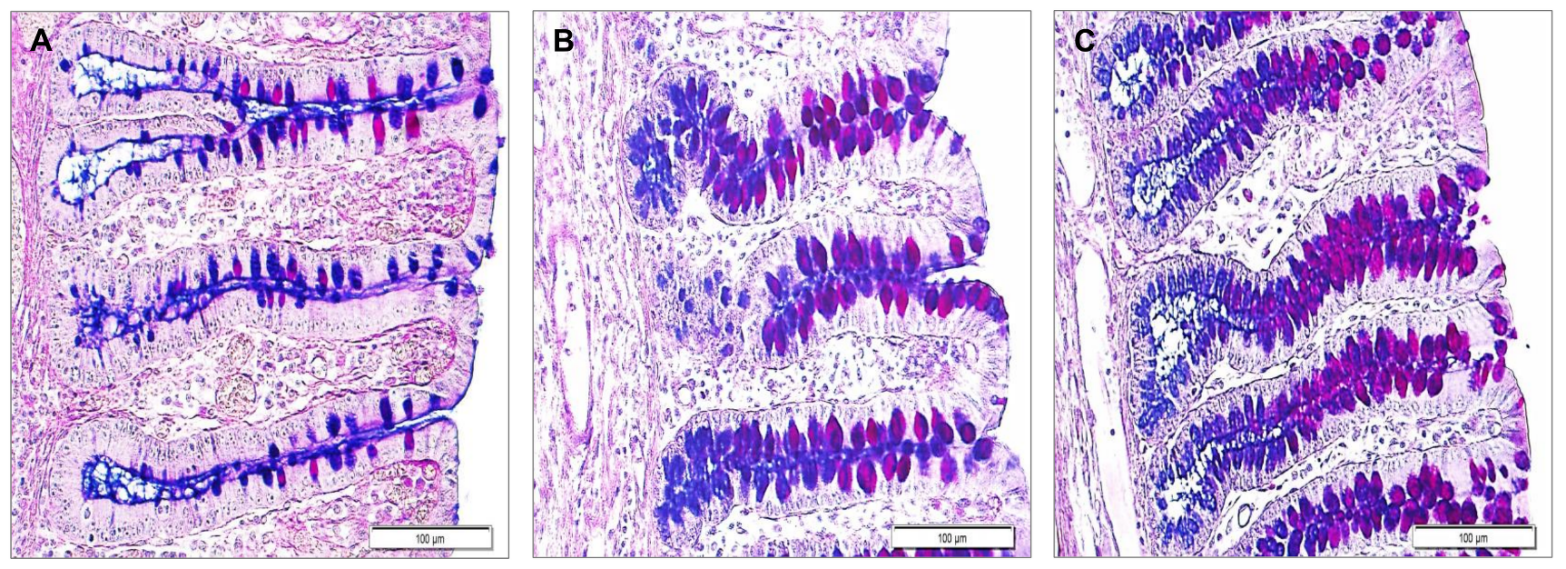
Alcian blue (pH 2.5)-periodic acid Schiff stained section in the ascending colon of weaned piglets. Mucin distribution and characteristics with three concentrations of dietary zinc treatments on 33 days of age in piglets. **A.** Low dietary zinc treatment (57 mg/kg zinc); **B.** Medium dietary zinc treatment (164 mg/kg zinc); **C**. High dietary zinc treatment (2425 mg/kg zinc), magnification X160. Neutral mucins (magenta) were found to be spread over the epithelial surface and the upper crypt, while acidic mucins (blue) dominated in the lower crypt area of the colon. The mixture of neutral-acidic mucins (magenta-purple or blue-purple colors) were mainly found along the crypt.

**Figure 2:**
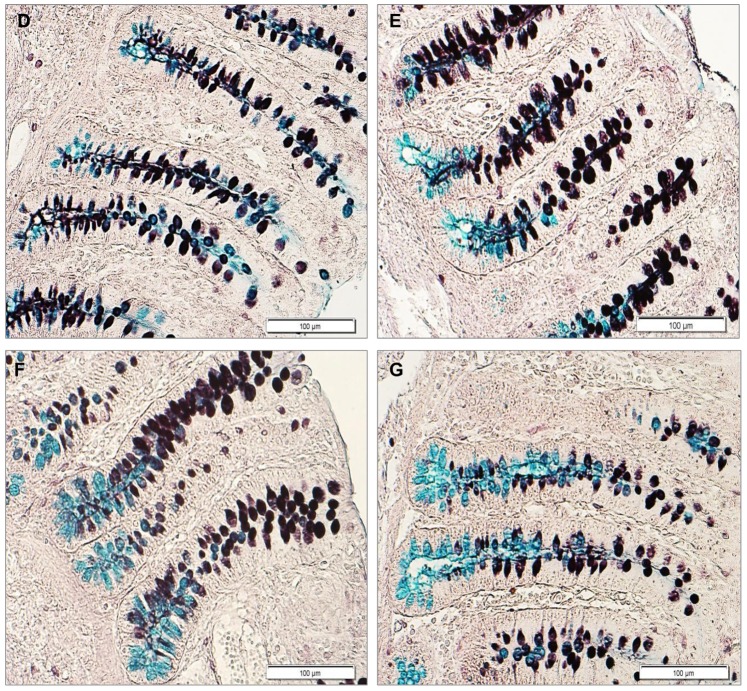
High iron diamine-Alcian blue (pH 2.5) stained section in the ascending colon of weaned piglets. Acidic mucins distribution and characteristics over 4 weeks post-weaning in piglets fed with medium dietary zinc treatment (164 mg/kg zinc). **D.** At 33 d of age; **E.** At 40 d of age; **F.** At 47 d of age; **G.** At 54 d of age; magnification X160. Secreting goblet cells with sulfomucins (black) dominated in the epithelial surface and the upper crypt, whereas goblet cells with sialomucins (blue) and mixed sulfo-sialomucins (black and blue colors) were located in the lower crypt of the colon.

With AB-PAS staining, goblet cells with neutral mucins were found to be spread close to the epithelial surface and in the upper crypt, while acidic mucins dominated in the lower crypt area of the colon. The mixture of neutral-acidic mucins was mainly found along the crypt (Figure 1). HID-positive cells with sulfomucins dominated in the epithelial surface and the upper crypt, whereas goblet cells with sialomucins and mixed sulfo-sialomucins were located in the lower crypt of the colon (Figure 2).

The chemotypes of colonic mucins changed mainly depending on zinc intake, but also on age (Table 4). High dietary zinc increased the amount of mixed neutral-acidic mucins (*P* = 0.019), and the total number of mucin producing goblet cells (*P* = 0.001). In addition, the older piglets had more goblet cells containing sialomucins (*P* = 0.043). There was no age or diet related effect on sulfomucins and mixed sulfo-sialomucins.

**Table 4:** Numbers of AB-PAS and HID-AB positive goblet cells per 1 mm basement membrane in crypts in the ascending colon of weaned piglets^1^.

Age	33 d			40 d			47 d			54 d				*P*-value		
Diet	LZn	MZn	HZn	LZn	MZn	HZn	LZn	MZn	HZn	LZn	MZn	HZn	SEM	Age	Diet	Age×Diet
AB-PAS staining																
Neu	4.8	3.1	3.0	2.6	3.1	2.8	4.7	2.8	3.7	3.1	3.4	4.3	0.24	0.535	0.490	0.444
Acid	22.1	20.9	24.0	20.8	21.1	23.1	22.0	24.7	26.5	21.9	28.0	25.4	0.64	0.196	0.155	0.725
NA	55.2	58.2	65.8	50.1	57.3	62.5	53.5	54.3	68.1	60.1	61.0	55.2	1.18	0.823	0.019	0.176
Total	62.1^ab^	61.0^ab^	68.9^a^	55.3^b^	60.8^ab^	65.7^ab^	60.8^ab^	62.1^ab^	73.6^a^	63.2^ab^	70.1^a^	64.9^ab^	0.82	0.088	0.001	0.099
HID-AB staining																
Sulfo	41.1	50.1	51.4	41.7	43.4	43.8	45.7	48.5	52.3	40.0	43.7	45.2	1.37	0.296	0.183	0.983
Sialo	3.6^ab^	1.1^b^	4.6^ab^	4.4^ab^	3.7^ab^	3.8^ab^	5.4^ab^	3.6^ab^	3.5^ab^	7.2^a^	4.9^ab^	6.0^ab^	0.37	0.043	0.130	0.813
Mixed	18.2	15.5	14.2	12.4	13.5	16.2	12.4	12.8	10.0	20.2	15.8	15.4	0.78	0.087	0.617	0.717

1 Ninety-six 26 day weaned Landrace piglets were randomly allocated into three diets with low, medium and high dietary zinc (57, 164, and 2425 mg/kg diet from ZnO). Eight piglets in each group were killed at the age of 33±1, 40±1, 47±1 and 54±1 d. Data were analyzed by two-way ANOVA followed by Tukey post hoc test. The data were given as mean values.

LZn: low dietary zinc; MZn: medium dietary zinc; HZn: high dietary zinc; AB-PAS: Alcian blue pH 2.5-periodic acid Schiff staining; HID-AB: high iron diamine-Alcian blue pH 2.5 staining; Neu: neutral mucin; Acid: acidic mucin; NA: mixed neutral and acidic mucins; Total: total number of AB-PAS positive goblet cells; Sulfo: sulfomucin; Sialo: sialomucin; Mixed: mixed sulfo-sialomucins.

ab Means with different superscripts within a row indicate significant differences between groups (*P*<0.05).

### mRNA Expression in the Colonic Tissue

The genes related to innate immunity and inflammatory cytokines were influenced by the dietary zinc concentration and age (Table 5). The expression of *MUC2* increased after 40 days of age, having the highest level at 47days of age (*P* = 0.040). The mRNA level of *TLR2* decreased until 47 days of age, and increased in the fourth week post-weaning (*P* = 0.031). The mRNA level of *TLR4* was affected by age and dietary zinc concentration. The expression of *TLR4* was down-regulated with age (*P* = 0.002), while its mRNA level with medium dietary zinc treatment was higher compared to the low dietary zinc group (*P* = 0.012). Moreover, an obvious dietary effect was found on the expression of *IL-8* (*P* = 0.015). The piglets fed high dietary zinc treatment had the lowest level of *IL-8* compared to those fed low and medium zinc supplementations, and the medium dietary zinc group had the highest expression.

**Table 5:** Relative gene expression (arbitrary values) of mucins and cytokines in the ascending colon of weaned piglets^1^.

Age	33 d			40 d			47 d			54 d				*P*-value		
Diet	LZn	MZn	HZn	LZn	MZn	HZn	LZn	MZn	HZn	LZn	MZn	HZn	SEM	Age	Diet	Age×Diet
MUC1	1.07	1.17	1.20	1.36	1.29	1.16	1.22	1.29	1.30	0.82	0.90	1.08	0.07	0.351	0.923	0.985
MUC2	0.55	0.49	0.61	0.56	0.36	0.59	0.93	0.71	0.73	0.83	0.78	0.74	0.04	0.040	0.459	0.946
MUC13	0.73	0.87	0.73	1.29	0.92	0.93	0.96	1.04	0.50	0.69	0.87	0.74	0.05	0.261	0.229	0.528
MUC20	0.74	1.37	1.89	1.43	0.75	1.39	1.31	1.18	0.58	1.11	1.13	0.90	0.11	0.732	0.954	0.196
TLR2	0.72	1.23	1.05	0.80	1.12	1.02	0.76	0.76	0.48	0.68	0.75	0.67	0.05	0.031	0.174	0.613
TLR4	0.89^a^	1.71^ab^	1.43^ab^	0.69^ab^	1.49^ab^	1.35^ab^	0.77^ab^	0.99^ab^	0.69^ab^	0.69^ab^	0.83^ab^	0.63^b^	0.07	0.002	0.012	0.500
IL-1β	0.30	1.18	1.62	0.64	0.67	0.50	0.81	2.00	0.81	2.03	3.32	0.64	0.28	0.345	0.327	0.690
IL-8	0.64	1.29	0.72	0.99	1.40	0.83	1.53	0.93	0.51	0.63	1.01	0.61	0.07	0.354	0.015	0.161
IL-10	0.47	1.76	1.46	0.79	1.39	1.98	1.82	1.75	1.31	1.89	2.31	0.78	0.20	0.845	0.483	0.553
IFN-γ	0.29	2.56	1.17	0.83	1.36	0.57	1.96	0.94	0.91	0.78	1.54	0.63	0.16	0.750	0.117	0.241
TGF-β	1.53	2.36	3.59	1.11	2.16	3.01	1.72	1.58	2.11	2.36	2.38	1.91	0.21	0.697	0.169	0.607

1 Ninety-six 26 day weaned Landrace piglets were randomly allocated into three diets with low, medium and high dietary zinc (57, 164, and 2425 mg/kg diet from ZnO). Eight piglets in each dietary diet were killed at age of 33±1, 40±1, 47±1 and 54±1 d. Data were performed by two-way ANOVA followed by Tukey post hoc test. The data were given as mean values in each group.

LZn: low dietary zinc; MZn: medium dietary zinc; HZn: high dietary zinc; MUC1: mucin 1; MUC2: mucin 2; MUC13: mucin 13; MUC20: mucin 20; TLR2: toll-like receptor 2; TLR4: toll-like receptor 4; IL-1β: interleukin 1β; IL-8: interleukin 8; IL-10: interleukin 10; IFN-γ: interferon-γ; TGF-β: transforming growth factor-β.

ab Means with different superscripts within a row indicate significant differences between groups (*P*<0.05).

No interaction was observed between age of piglets and zinc concentration in parameters related to morphometric characteristics, mucin composition and gene expression.

## Discussion

The influence of age and dietary zinc level on the morphology, the composition of mucin and immunological traits in the colon of weaned piglets has not been fully elucidated. The present study showed that three different dietary zinc levels, covering a broad range from marginally low to pharmacologically high concentrations, have led to significant effects on various parameters in colonic tissue. All diets contained enough zinc to cover the recommended dietary levels [23]. Feed intake, feed conversion and growth rate of the piglets were not affected by the different dietary zinc concentrations in the first two weeks of the experiment, however, weight development of the high dietary zinc group were reduced after three weeks of the experiment [17]. Amongst dietary effects, a substantial part of the observed changes in the measurements was also age-dependent. Interestingly, in addition to the morphometric measurements that were affected by dietary zinc concentration and age of piglets, there were also significant effects regarding the expression of biomarkers relevant for the innate immune system and inflammatory response.

Crypt depth was affected neither by the dietary zinc intake nor age, but the crypt area increased between 33 and 54 days of age. Interestingly, the highest dimensions were obtained with the medium dietary zinc group. So far, only few data are available on the morphology of the colon in pigs in response to dietary factors. The lack of dietary effects on the crypt depth may be partly explained by the fact that even the low dietary zinc diet (57 mg/kg diet) was sufficient for the constant renewal of the colonic crypts. Generally, the lowest dosage of zinc in this study practically corresponds to a marginal-adequate supply situation, according to NRC recommendation [23]. Due to the expected high priority of nutrient partition for intestinal cell growth and renewal, it can be assumed that the proliferative activity of colonocytes was maintained even at suboptimal supply situations. In rats, it was shown that very low dietary zinc intakes induced a lower proliferative activity of mucosal epithelial cells [24] and colonocytes [25]. Regarding the crypt area, an age effect became apparent, older animals having greater crypt areas. This could probably be explained by the growth in body size and ongoing maturation of the intestinal tract during the post-weaning period. The highest surface areas were observed in the medium dietary zinc group, while the high dietary zinc intake resulted in the lowest surface area and the low concentration of dietary zinc resulted in intermediate values. This suggests that there was a relevant physiological effect on the tissue structure between the low and medium dietary zinc concentration, while the high dietary zinc treatment did not lead to further reactions. This could, in view of the high zinc content in the digesta, indicate that very high zinc supplement in the diet lead to a reversal effect on cell growth in the colon. With regard to the morphological data of the colonic tissue, there is relatively little comparative information on the impact of zinc or other trace elements in pigs. High dietary zinc intakes were shown to have no significant effect on crypt depth in the large intestine of pigs [26], confirming our data. Other dietary factors, such as protein intake [27], hydrolyzed straw meal [28], butyrate [29], fructooligosaccharides [30], potato protein, and cellulose [31] were shown to increase crypt depth in the colon of pigs. A study focusing on the effects of different fractions of grain kernels found only minor changes on crypt depth in the large intestine of pigs across treatments [32]. Spray dried porcine plasma had an impact on immune cells in the tissue, however, it did not affect colonic crypt depth [33]. Coarse ground corn, sugar beet pulp and wheat bran also had no impact on this parameter [34]. The data on crypt depth in the referenced studies were similar to data in the present study. If and how the colonic surface area can affect the absorptive capacity of the large intestine for electrolytes and water [27], needs further characterization in piglets.

Mucins are an important protective barrier within the gastrointestinal tract. The mucin layer safeguards the intestine and its structural integrity. A considerable part of the formed mucins is secreted into the intestinal tract and used as nutrient source for the resident microbial communities [35]. Furthermore, the intestinal lining serves as binding site for various gut wall-associated microorganisms, namely lactobacilli [36], [37] but also pathogens such as salmonellae [38]. Mucins can be broadly categorized into neutral and acidic chemotypes, and the latter are further divided into sialomucins and sulfomucins based on the presence of the respective terminal acids in the oligosaccharide chain [39]. It has been well documented that sulfomucin abundance was greater than sialomucin production in colonic mucosa, both in humans [40] and pigs [41]. This staining characteristic was also observed in the present study. The goblet cells with sulfomucins were mainly distributed in the upper crypt and surface epithelium, whereas sialomucins were presented in the lower crypt area of the ascending colon. Reverse distributions have been shown in humans [40]. This may be due to species differences, but also due to age effects and the studied segment of the large intestine. Moreover, the distribution of neutral, acidic and mixed neutral-acidic mucins was similar in a previous study in pigs [41]. Mucin chemotypes in the colonic goblet cells were affected by both, age and zinc intake in our study. Older piglets had a higher density of sialomucin producing goblet cells, indicating a qualitative maturation process in the 4-week experimental period. Therefore, age seems to have a strong impact on mucin composition in piglets. The colonic mucins are highly sulfated and sialylated in new born pigs [42]. In the colon, no major changes of mucin chemotypes occurred in piglets 1–13 d after weaning on a soy based diet [41]. High dietary zinc treatment increased the amount of neutral-acidic mucins and the total number of mucin containing goblet cells in this study. This is confirming former studies, where high dietary zinc enlarged the total area of mucin staining in the cecum and in the colon of young piglets [26]. Speculatively, this might result in a better protection against diarrhea and pathogen invasion in the post-weaning period of piglets. It also has been shown that several other feed ingredients influenced the formation and composition of mucins in the colonic mucosa [43]. In particular, physical factors of the diet are considered as important. A coarser particle structure and a change in the viscosity with diets containing carboxymethylcellulose changed the number of goblet cells and mucins and were considered as indicators of gut maturation [38], [44].

The interaction between age and dietary factors and the expression of various mucin types are considered increasingly relevant, as mucins are important as first barrier against invading bacteria. In the present study it was shown that the expression of the *MUC2* gene increased age-dependently. Apparently, no effect was caused by the varying zinc intake. *MUC2* is mainly responsible for the formation of the gel properties of the intestinal mucus [45]. A lack of mucin 2 or changes in the tissue specific glycosylation lead to a predisposition of diseases in humans such as colitis and colon cancer [46], [47]. A fault in the corresponding mucus formation and also a change in the glycosylation of mucins can lead to disorders of colonic function and health impairment. Zinc is clearly related to mucin formation, as *MUC2* expression is regulated by the zinc-finger *GATA-4* transcription factor in intestinal cells [48] and *ZIP4* knockout mice exhibited abnormalities in mucin accumulation in *Paneth* cells [49]. Obviously, all diets in the present study provided sufficient zinc for the *MUC2* gene expression. In pigs, preterm conditions have been shown to impair *MUC2* synthesis, predisposing young piglets to develop necrotizing enterocolitis [50]. Colonic mucin gene expression was influenced by the inclusion of laminarin in the diet [51] while there was no effect of low digestible dietary protein and fermentable carbohydrates [52]. *MUC13* belongs to the most abundant *MUC* genes in the gastrointestinal tract. Piglets fed a diet with faba beans had a higher *MUC13* expression in the intestinal segments perfused with a faba bean suspension [53]. *MUC1* and *MUC20* are as well important for the intestinal mucus formation, however, also here only few studies determined the effects of nutritional factors on mucin gene expression in the intestinal tract of pigs. *MUC1* is a large transmembrane glycoprotein expressed on the apical surface of the majority of reproductive tract epithelia [54], whereas the porcine *MUC20* gene is associated with susceptibility to enterotoxigenic *Escherichia coli* F4ab/ac [55].

The luminal mucin forms the first barrier against pathogen invasion, the epithelium and the lamina propria are the second line of host defense. They sense the invading pathogens by recognizing pathogen-associated molecular patterns (PAMPs) via pattern recognition receptors (PRRs), and TLRs are one group of PRRs in innate immunity. TLRs can activate a common signaling pathway leading to the activation of mitogen-activated protein kinase and nuclear translocation of transcription factor NF-κΒ, which activate immune cell response and lead to production of inflammatory cytokines and co-stimulatory molecules [56]. There are 13 known members of mammalian *TLRs. TLR2* and *4* are expressed in various lymphoid tissues of the porcine intestinal tract and play an important role in innate immunity in the young pigs [57], [58]. However, only limited data showed that dietary factors, such as yeast extracts [59] and a fish oil supplement [60] reduced the expressions of *TLR2* and *TLR4* in pig intestine. In the present study, compared to medium dietary zinc supplementation, sub-optimal levels of dietary zinc treatments down-regulated the expression level of *TLR4*. Moreover, we also found that the expressions of *TLR2* and *TLR4* were down-regulated in our piglets with age, which is in accordance with previous findings indicating that *TLRs* were regulated by age of pigs [61], [62]. Weaning induces transient gut inflammation in piglets [63] and the expression levels of some pro-inflammatory cytokines such as *IL-1β*, *IL-6* and *TNF-α* were up-regulated in newly weaned piglets [64]. Over-production of pro-inflammatory cytokines usually results in impaired intestinal integrity and epithelial function [65]. Controlling the expression level of pro-inflammatory cytokines may have potential benefits in alleviating gut mucosal inflammation and reducing the incidence of diarrhea [66]. Zinc has been well documented to show a beneficial role on inflammatory lesions [67]. In some recent studies, mRNA levels of *TNF-α*, *IL-6* and *IFN-γ* decreased with increasing concentrations of dietary zinc in pigs [68], [69]. *IL-8* is a signal protein, which is essential for neutrophil recruitment and seems to be of importance in establishing protective immunity [70]. Normally barely detectable in healthy tissues, it is rapidly induced by 10 to100-fold in response to pro-inflammatory cytokines and the variation of expression is one of the remarkable properties of *IL-8* [71]. *In vitro*, zinc oxide counteracted the expression of the inflammatory *IL-8* level caused by enterotoxigenic *Escherichia coli* [72]. Accordingly, we also found that the mRNA level of *IL-8* was down-regulated with the high concentration of dietary zinc treatment. *TGF-β* and *IL-10* are anti-inflammatory cytokines and can protect the intestinal barrier function. The mRNA levels of *TGF-β* and *IL-10* were increased with high zinc oxide supported on zeolite on day 7 post-weaning [69]. However, weaning induced transient increase of inflammatory cytokines for 2 days, then most of cytokines rapidly decreased to the pre-weaning levels after day 9 post-weaning [64].

## Conclusions

The present study revealed that high levels of dietary zinc oxide had specific effects on the colonic morphology, mucin composition and expression of genes related to innate immunity and inflammatory processes in weaned piglets. The findings suggest a positive impact on the maturation of the barrier function of the colonic mucosa, displayed by an increase in mucin producing goblet cells and a down-regulation of *IL-8* and *TLR-4*. The observed changes suggest that high dietary zinc dosages stimulate protective mechanisms in colonic function which may help to understand the protective mode of action of very high dietary levels of zinc oxide against the commonly occurring post-weaning diarrhea in piglets.
